# Comparison of Construction Strategies of Solid Electrolyte Interface (SEI) in Li Battery and Mg Battery—A Review

**DOI:** 10.3390/molecules29194761

**Published:** 2024-10-08

**Authors:** Zhongting Wang, Rongrui Deng, Yumei Wang, Fusheng Pan

**Affiliations:** 1 National Engineering Research Center for Magnesium Alloys, College of Materials Science and Engineering, Chongqing University, Chongqing 400044, China; wang.zhongting@cqu.edu.cn (Z.W.); fspan@cqu.edu.cn (F.P.); 2 National University of Singapore (Chongqing) Research Institute, Chongqing 401123, China; rongrui.deng@nusricq.cn

**Keywords:** solid electrolyte interface (SEI), lithium-ion battery, magnesium-ion battery

## Abstract

The solid electrolyte interface (SEI) plays a critical role in determining the performance, stability, and longevity of batteries. This review comprehensively compares the construction strategies of the SEI in Li and Mg batteries, focusing on the differences and similarities in their formation, composition, and functionality. The SEI in Li batteries is well-studied, with established strategies that leverage organic and inorganic components to enhance ion diffusion and mitigate side reactions. In contrast, the development of the SEI in Mg batteries is still in its initial stages, facing significant challenges such as severe passivation and slower ion kinetics due to the divalent nature of magnesium ions. This review highlights various approaches to engineering SEIs in both battery systems, including electrolyte optimization, additives, and surface modifications. Furthermore, it discusses the impact of these strategies on electrochemical performance, cycle life, and safety. The comparison provides insights into the underlying mechanisms, challenges, and future directions for SEI research.

## 1. Introduction

Renewable energy has been rapidly adopted in recent years to relieve the emissions of CO_2_ all over the world. However, renewable energy heavily depends on natural resources, such as sunlight, wind, water and so on, which are unpredictable and out of human control. Therefore, renewable energy is stored and then used in a controlled manner. Electrochemical energy storage systems, which are based on storing chemical energy and converting to electrical energy when needed, are the most traditional energy storage devices for power generation. Rechargeable batteries are one of the oldest and also one of the most widely used electrochemical energy storage systems.

Lithium-ion batteries have dominated the commercial battery market since the 1990s, including in aerospace, transport and electronics, due to their high gravimetric and volumetric energy density. Other than lithium-ion batteries, next-generation rechargeable battery technologies are also in development. Besides alkali metal-ion batteries, other metals are also considered as anodes in rechargeable batteries. For example, magnesium has a very high theoretical volumetric energy density—around 50% higher than that of lithium. Moreover, magnesium anodes do not exhibit dendrite formation, albeit only in certain nonaqueous solvents and at certain current densities. This allows magnesium metal to be used at the anode with around a five times higher volumetric energy density than a graphite electrode. The magnesium’s relative abundance and ease of mining make magnesium-ion batteries good candidates for large-scale energy storage [1].

**Figure 1:**
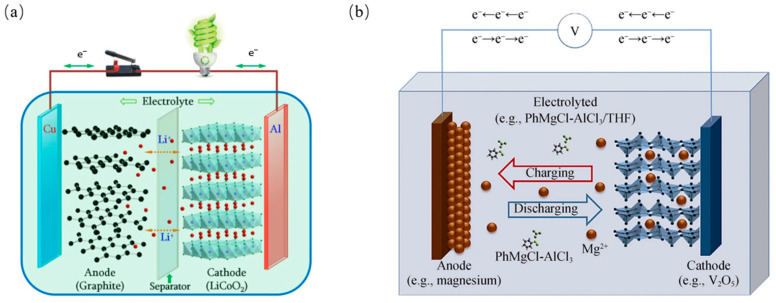
Working principle of (**a**) Lithium-ion battery (**b**) Magnesium-ion battery [2,3].

The working principle of these batteries is similar. As shown in Figure 1, during the discharge process, the oxidation and reduction reactions happen on the anode and cathode, respectively, while the metal ions move from the anode to the cathode through electrolytes (vice versa during the charge process) [2,3]. The electrode/electrolyte interface is obviously an important electrochemical juncture that determines the behaviors of metal ions and electrons. Although the electrolytes in batteries are designed to be stable with a wide electrochemical window, the kinetic stability between electrolytes and electrodes is attained after a stable solid electrolyte interface (SEI) forms. The SEI is the product, where trace amounts of electrolyte decompose and react with electrodes. An ideal SEI film should inhibit further electrolyte degradation and electron transport while facilitating ion transport.

In recent decades, many researchers have spent huge amounts of time trying to figure out the exact formation, structure and functional mechanisms of the SEI. However, these questions remain the most ambiguous issues in battery science. The thickness of the SEI is around 10–15 nm, containing both organic and inorganic species, which is the result of a delicate balance between electrolyte decomposition and passivation. The thickness is controlled by several factors, including the composition of the electrolyte, the applied current density, and the anode surface properties. A thinner SEI might not provide sufficient protection against side reactions, leading to reduced cycling stability, while a thicker SEI could impede ion transport, adversely affecting battery performance [4,5]. Therefore, this range is optimal for maintaining a balance between protection and ion conductivity. In addition, the formation and growth of the SEI occurs during the charging/discharging processes. These characteristics make it very hard to investigate SEIs using traditional real-time in situ characterization technology. To further improve the capacity of current commercial batteries and the development of next-generation batteries, the design of the SEI is an unavoidable challenge. Specially, the Li metal battery is considered the ‘holy grail’ of batteries due to its extremely high capacity. One of the greatest obstacles of Li metal batteries is that it is difficult to construct a stable SEI film on an Li metal surface, which not only degrades the capacity after charging/discharging cycles but also causes the hazards of flammability and explosiveness. Moreover, in other battery systems, such as magnesium-ion batteries, the ideal anode materials are also the corresponding metals. Therefore, these batteries also face the same challenge: a stable and ion-conductive SEI film.

However, although the properties of alkali elements and alkaline elements are very similar, the slight difference makes a significant difference to the interface configuration. Since dendrite is the main reason for fires and explosions, the focus of SEI configuration in lithium-ion batteries (LIBs) is not to stabilize the interface but to also suppress the formation of dendrite. For sodium-ion batteries (NIBs), although sodium is a monovalent ion like lithium, its larger ionic radius leads to greater volume changes during cycling. This can cause the SEI in NIBs to crack and reform, making it less stable over time. The SEI tends to be thicker and less uniform compared to LIBs, and while Na_2_CO_3_ and NaF are formed similarly to their lithium counterparts, the mechanical stresses in NIBs due to the size of Na^+^ present additional challenges. Similar to NIBs, the SEI in potassium-ion batteries (KIBs) forms through electrolyte decomposition, but the larger size of potassium ions can complicate the formation mechanism and composition. The stability and ionic conductivity of the SEI are often lower than those in LIBs and NIBs, potentially limiting the long-term performance of KIBs. On the other hand, now, the passive film on the anode is the main bottleneck in magnesium-ion batteries (MIBs); hence, the SEI configuration in MIBs mainly focuses on constructing a stable interface with high ionic conductivity. Calcium-ion batteries (CIBs) face similar challenges: the SEI should provide ionic conductivity and prevent the direct reaction of the calcium metal with the electrolyte, which can lead to unwanted side reactions and decreased battery efficiency. Hence, we find that the SEI functions of alkali metal-ion batteries and alkaline earth metal-ion batteries are not the same, so we selected the SEIs of lithium-ion batteries and magnesium-ion batteries as representatives for discussion.

This review will summarize SEI formation and the influence on the electrode/electrolyte interfaces, as well as compare the different configurations between LIBs and MIBs. Finally, perspectives on the investigation of SEI films are also discussed, including computer simulations and experiments.

## 2. The Formation Mechanism of SEIs

In 1970, Dey [6] first observed the passive film on the Li metal surface, and Peled [7] first proposed the concept of the solid electrolyte interface (SEI) in 1979. In 1985, Muller [8] firstly identified the existence of Li_2_Co_3_ and polymers in SEIs by X-ray diffraction, which illustrates the presence of both inorganic and organic components in SEIs. In 1990, Dahn [9] reported the gradual formation of an SEI on graphite anodes. In 1995, Kanamura [10] found that a thin, dense LiF film is attached on the Li anode surface, and another porous composite layer is above this film. This study inspired the design of components for artificial SEI films. In 1997, Peled [11] developed the mosaic model for the SEI, which considers the grain boundaries. In 1999, Aurbach [12] combined ex situ Fourier-transform infrared spectroscopy (FTIR) and in situ atomic force microscopy (AFM) to investigate the formation process of the SEI. In the 21st century, researchers began to use computer simulations and advanced characterization techniques to accelerate the study of SEIs. In 2001, Balbuena [13] utilized density functional theory to study the reduction mechanism of electrolytes on anodes, which also illustrated the formation mechanism of the SEI. In 2010, Xu [14] quantitatively measured the energy barrier for Li ions to pass through the SEI. In 2017, Cui [15] observed mosaic structure of the SEI film by cryo-electron microscopy. In 2022, Song [16] investigated the chemical composition of the SEI by time-of-flight secondary ion mass spectrometry (TOF-SIMS).

**Figure 2:**
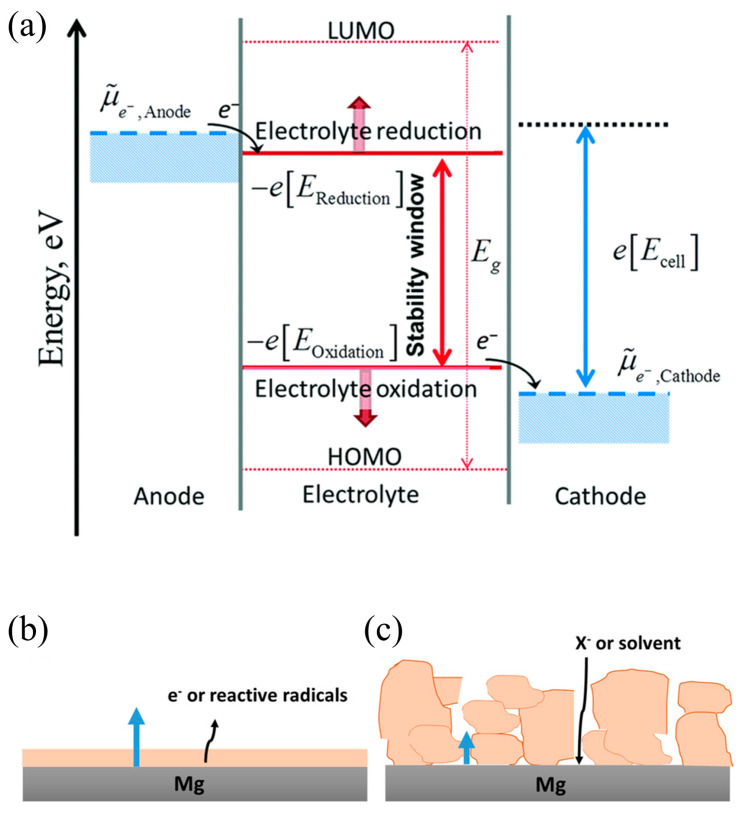
(**a**) Schematic illustration of the positive and negative potential limits of electrolyte stability, and the energy levels of LUMO and HOMO [17,18]. The red arrow represents stability window, the pink arrow represents the energy difference between LUMO and HOMO, the blue arrow represents the electrochemical potential difference between cathode and anode. Mechanisms of SEI growth on Mg electrode: (**b**) dense film growth by electronic or magnesium interstitial conduction and (**c**) porous film growth by anion or solvent diffusion through the pores. In both cases, growth occurs at the metal/SEI interface [24].

At present, molecular orbital theory is the general explanation of the SEI formation mechanism in LIBs, as shown in Figure 2 [17,18]. The theory explains that when the lowest unoccupied molecular orbital (LUMO) of the electrolyte is lower than the electrochemical potential of the anode (μ_A_), the electrolyte will gain electrons from the anode and is reduced, forming an SEI film on the anode surface. On the other hand, when the highest occupied molecular orbital (HOMO) of the electrolyte is higher than the electrochemical potential of the cathode (μ_C_), the electrolyte will lose electrons and is oxidized, forming a CEI (cathode electrolyte interface) film on the cathode surface. The SEI film then will expand the electrochemical window of the electrolyte, hindering further reactions and preventing the decomposition of the organic electrolyte.

It is believed that the formation mechanism of the SEI in Mg ions also follows Peled’s model, in which the SEI is considered to be an inorganic/organic nanocomposite material, and the ion transport through the bulk inorganic part is the rate-determining step [19]. However, the low ionic conductivity of inorganic compounds, such as MgO, MgF_2_, and MgS, and the Pilling–Bedworth (P–B) ratios (ratio of the volume of the elementary cell of a compound and the elementary Mg cell) are dissimilar, pointing toward the formation of a porous surface layer on the Mg. In a recent study, anion adsorption from the electrolyte on the surface of such Mg-SEIs was suggested, which fully blocks the cation transport [20].

Two scenarios of continuous SEI growth on Mg are thus considered, as shown in Figure 1. In the first scenario, the growth proceeds because of the electronic conductivity and/or reactive radical existence in the thin film formed upon initial Mg contact with the electrolyte [21]. In the second, long-term growth occurs due to the decomposition of the anion and/or solvent molecules, which arrive at the Mg/SEI interface by diffusion through the SEI pores [22,23]. Electronic tunneling is disregarded as the SEI thickness is expected to be higher than several nm after a longer cell rest time. In both cases, the amount of SEI material typically increases over time [23].

## 3. SEI in Li-Ion Batteries

Researchers have illustrated that the initial SEI forms during the charging process in the early cycles. At the early stage, the SEI hinders the transportation of solvent molecules and lithium salt. Therefore, the SEI continues to grow at this stage.

The well-known single-electron reduction reaction, which explains the organic by-products, happens at this stage. During the reaction, the nucleophilic interactions between radical anions formed by electron addition to the carbonate molecules, assisted by the vicinity of lithium ions and carbonyl group, occurs and generates lithium salts of semi-carbonates or alkyl carbonates [25]. It is found that ethylene and propylene are the gaseous side-reaction products in the reduction, attributed to the decomposition of ethylene carbonate (EC) and propylene carbonate (PC), respectively, while alkanes are the reduction side-reaction product of linear carbonates through the single-electron reaction mechanism [26,27,28,29,30,31,32]. Besides this mechanism, Collins proposed the two-electron reaction mechanism, which also explains the alkyl carbonates and gaseous side-reaction products [33]. Besides the lithium alkyl carbonates mentioned above, oxalates and succinates are also SEI components in the SEI in EC/PC or EC/DMC electrolytes, where DMC is the abbreviation for dimethyl carbonate [34,35]. Moreover, acetals and ortho-esters are also found in the SEI by NMR through various formative mechanisms [36].

On the other hand, the typical inorganic products, including LiF, LiCl and Li_2_O, precipitate on the electrode surface through a reduction reaction [19]. Low-abundance CO_2_ is also detected during the formation of the SEI [37]. CO instead of CO_2_ is also reported as a by-product [38,39,40,41]. Li_2_CO_3_ is also reported, due to the elimination of the reaction of Li_2_O with CO_2_, in inefficient moisture elimination cells [42,43,44].

In addition to small molecules, polymers and oligomers are also involved in SEI composition [30,38,45,46]. Long-chain structures are reported in EC/DMC and PC electrolyte systems [47,48]. Compared with short-chain structures, polymer species could provide better protection function, and the branched polymer with carbonate/ethylene oxide units could assist the lithium ions in passing through the layer [49]. However, it is still ambiguous whether the polymer component and semi-carbonates are partially soluble, which affects the stability of the SEI [19].

After long cycles, the initial SEI would evolve and change gradually [50,51,52]. The unstable species growth at the initial stage in the SEI stops, and new stable products, such as inorganic compounds, tend to form [53,54]. The local dissolution or the uneven stress will also cause the mechanical breakdown of the SEI. In the case of fast-forming cracks, the electrolyte flows into the crack and immediately reacts with the fresh anode surface, forming a new thin protective film and preventing further local reactions. On the other hand, in the case of slow-forming cracks, the SEI becomes thinner and electrons pass through the thin region and reduce the electrolyte further [19].

Some models propose that inorganic species such as Li_2_O, LiF and Li_2_CO_3_ tend to attach on the surface of electrode, while organic species tend to exist close to the electrolyte [55,56]. The study also shows that the inorganic section has higher conductivity than the organic section [55], which implies that during the evolution stage, the conductivity of the SEI tends to increase.

Gas is also detected during the evolution stage, which is believed to be one of the reasons for capacity fading [57]. The crossover reactions on the cathodic side can generate products like CO_2_, which can diffuse inside the anode and destabilize the SEI, as well as be reduced into Li_2_CO_3_, leading the evolution of the SEI [58].

**Figure 3:**
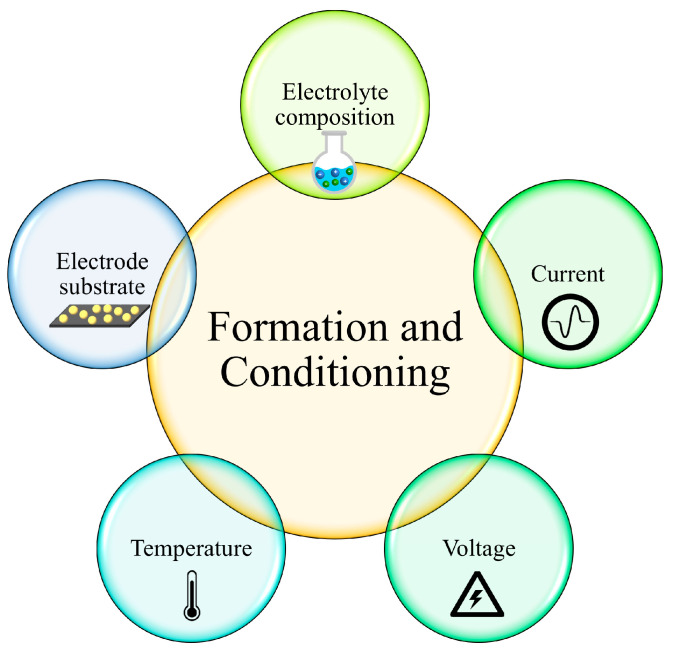
Factors affecting the formation of the SEI layer [19,59,60].

During the formation of the SEI, several factors affect the properties of the SEI layer, as shown in Figure 3 [19,59,60]. Firstly, the composition of the electrolyte is one of the key factors that determines the SEI’s features [61]. For example, the weak interaction between solvents could prevent the exfoliation of graphite. EC, which is one of the most common solvents with low lithium binding energy, can participate in SEI formation, instead of co-intercalating into graphite [9,62,63,64,65]. On the other hand, PC, another commonly used solvent, tends to co-intercalate into graphite layers and make graphite exfoliate [66]. This phenomenon proved that the difference of a single methyl group between EC and PC would significantly change the SEI composition and affect the behavior of Li ions [67,68,69]. The functional groups of electrolyte molecules influence the precursor constituents and resultant reactivity. It is also found that increasing the lithium salt concentration in the electrolyte could weaken the Li^+^ ion solvation sheath and consequently protect graphite against exfoliation [70]. Moreover, the solvents also affect the composition of the SEI. The PC-based electrolyte also promotes the formation of Li_2_CO_3_ in the SEI, while EC-based electrolytes promote the mixture of semi-carbonates, oxalates and oligoethers [71].

Besides the solvents, the type of lithium salt also affects the features of the SEI. Li_2_CO_3_ was the main SEI component in lithium bis(trifluoromethane)sulfonimide (LiTFSI) salt electrolytes, while the main component in LiBETI salt electrolytes was a mixture of Li_2_CO_3_ and semi-carbonates [27]. This is because the decomposition of anions varies significantly [72,73]. LiPF6 would cause the formation of Li_x_PF_y_ and Li_x_PF_y_O_z_. LiBF_4_ would lead the formation of Li_x_BF_y_. Lithium difluoro (oxalate) borate (LiDFOB) would form lithium oxalates, and LiFSI causes the formation of Li_3_N(SO_2_)_2_ in the SEI [25].

As the substrate of the SEI, the electrode also an important factor for determining the SEI’s features. The particle size, pore size, and degree of crystallinity of the electrode all affect the formation of the SEI, which influences the processes of industrial production [74,75,76]. The small particles provide more edge sites, which have higher energy and promote the formation of the SEI. Therefore, the thickness of the SEI is greater in the cross-sectional planes than the basal planes [77]. The oxygen groups on the graphite surface would reduce the potential and promote the formation of the SEI before the intercalation. These groups are also usually at the edge sites, where lithium needs to pass though before interaction. Therefore, the ratio of the edge planes to the basal planes determines the electrochemical performance of graphite in SEI formation [77,78]. Moreover, the components of the SEI at the edge are different from those of the SEI at the basal planes. More inorganic compounds like LiF are in the SEI at the edge sites, and more organic compounds are in the SEI at the basal planes [79].

Apart from the properties of the material, the external condition also affects SEI formation. The applied current rate, the current density, the state of charge and the temperature are the main external factors [51,78,80]. High current rates can accelerate the growth of the SEI and consequently cause significant capacity loss [81]. On the other hand, increasing the current density at the electrode then can relieve the capacity loss [19]. A high state of charge and overcharging will cause a change in SEI structure and capacity fade [51]. A high operation temperature will increase the thickness of the SEI and cause thermally unstable components to dissolve [82].

Normally, the intrinsic SEI has poor performance and causes capacity fade, especially in lithium metal batteries. Then, there are two ways to enhance the performance of the SEI. One is to enhance the inherent properties of the SEI, and the other is to construct an artificial SEI. To enhance the inherent properties of the SEI, modifications can be applied on the electrolyte and the electrode.

The modification of electrolytes is the most commonly used method to improve the intrinsic SEI [18]. The strategy of electrolyte modification is to change the Li^+^ solvation structure. The anions then become more involved in the solvation structure, forming more contact ion pairs (CIPs) and aggregates (AGGs). Therefore, a more stable SEI is constructed, which can improve the columbic efficiency of the battery [83]. There are three methods to modify the electrolyte: modifying the solvent, modifying the lithium salts and adding additives.

**Figure 4:**
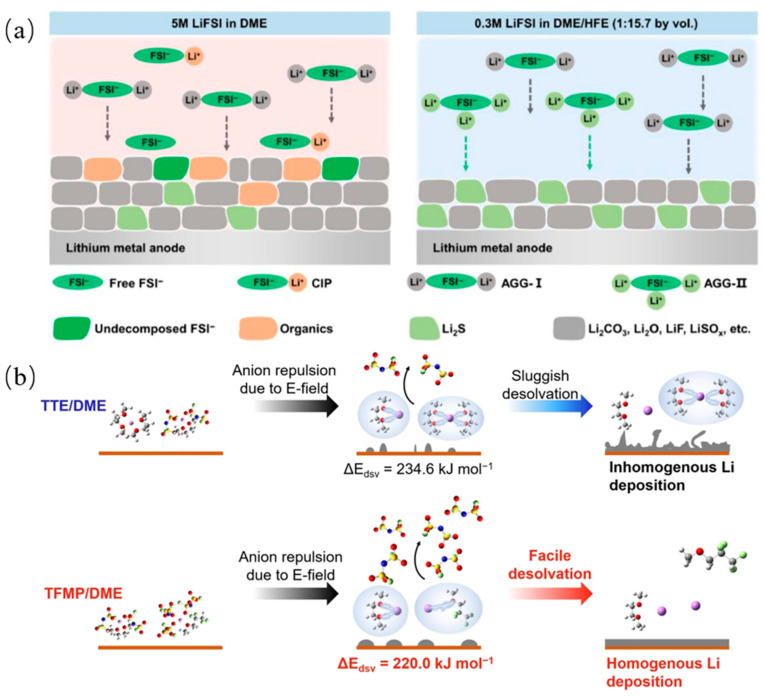
(**a**) Schematic diagram of solvent sheath structures of FSI^−^ anion in 0.3 M LiFSI in DME/HFE and 5 M LiFSI in DME electrolytes and the generated component in SEI films [84]. (**b**) Desolvation mechanism and corresponding desolvation energy obtained from MD simulation and DFT calculation of 1 m LiFSI-TTE/DME localized high concentration electrolyte and 1 M LiFSI-TFMP/DME electrolyte [86].

The solvent affects the Li^+^ solvent sheath structure, which in turn determines the SEI components and structure, as discussed previously. By adding 1,1,2,2-tetrafluoroethyl-2,2,3,3-tetrafluoropropyl ether (HFE) into a DME solvent, since HFE does not participate in the Li^+^ solvent sheath structure when diluting the electrolyte, the decomposition of the anions in the sheath structure is promoted, resulting in the formation of inorganic-rich SEI, as shown in Figure 4a [84]. The development of all fluorinated electrolytes has wide LUMO-HOMO gaps in the solvent, and the formed LiF-rich SEI has a columbic efficiency of 97.1% [85]. The 1,1,2,2-tetrafluoro-3-methoxypropane (TFMP) solvent will induce the formation of a core–shell-like solvation structure of the electrolytes and exhibit more AGGs, as shown in Figure 4b [86]. This solvent will also effectively improve the ionic conductivity, reduce the solvation energy, and stabilize the SEI layer. Another fluorinated ether solvent, 1,1,1-trifluoro-2,3-dimethoxypropane (TFDMP), is also used and helps the electrolyte to achieve high ionic conductivity (7.4 mScm-1) and antioxidant properties (4.8 V) [87].

Lithium salts are another effective method to improve an SEI—for example, increasing the lithium salt concentration to form more CIPs and AGGs. It is reported that by increasing the concentration of LiFSI in the carbonate electrolyte, a stable LiF-rich SEI forms with high Coulomb efficiency (CE) (99.3%) [88]. LiNO_3_, as the only lithium salt, combined with an FEC co-solvent can construct a stable Li_3_N–LiF-rich SEI layer with a high CE of 98.31% [84].

Instead of changing solvent and salts, adding additives into mature electrolytes is a more convenient way to improve the intrinsic properties of electrolytes. Fluorinated additives are commonly used to form an LiF-rich SEI. The fluoroethylene carbonate (FEC) additives can produce an LiF-rich SEI, which promotes the uniform deposition of lithium ions [89]. However, HF gas is produced by the decomposition of FEC, which is detrimental to health [18]. Another fluorinated additive, LiDFOB, was synthesized and added into ether electrolytes. After adding LiDFOB, an LiF-rich SEI will form with an organic matrix due to the ring-opening polymerization of DOL. The organic–inorganic composite SEI endows LIBs with superior performance [90]. Surprisingly, protein can also be used as the additive. A natural protein from zein was added into electrolytes. As the result, a variety of polar functional groups, such as -COOH, -NH_2_, and -OH, were introduced into the electrolyte, participated in the formation of the SEI, and formed an organic-rich SEI. This organic-rich SEI can help lithium deposit more uniformly and repair the SEI crack induced by the volume change of the anode [91].

Since one main reason for SEI breakup is due to the volume change of the electrode, modifying the electrode is another efficient way to enhance the inherent properties of the SEI. The fabrication of a lithium composite anode with no volume expansion by roll-to-roll can eliminate dendrite formation and help lithium ions uniformly deposit [92]. A sandwich structure anode is designed with high energy density, consisting of an insulating layer (a mixture of polyvinylidene fluorideco-hexafluoropropylene and LiNO_3_), a mesoporous layer (a Cu-coated carbon fiber matrix), and a lithium-friendly layer (LiMg) in a roll-to-roll way. This sandwich anode can help lithium ions bottom-up deposit [93]. Also, 2D structured MXene nanosheets (Ti_3_C_2_T_x_) were designed to promote the uniform growth of lithium. The fluorine end groups from MXene lead to a homogeneous distribution of LiF in the SEI [94]. Furthermore, 2D titanium carbonitride (Ti_3_CNT_x_) and 3D reduced graphene oxide (rGO) frameworks as Li scaffolds were designed to guide the formation of the SEI. The –F group of Ti_3_CNT_x_ promotes the decomposition of LiTFSI to promote the formation of an LiF-rich surface. The SEI layer has an ordered layered Li_2_O shell with internal LiF nanoparticles [95].

Enhancing SEI performance through material modification is more or less limited by the inherent properties of the material. Therefore, constructing an artificial SEI seems another good method to form an ideal interface.

The purpose of an artificial SEI is not only to protect the surface of electrode, but also to suppress dendrite growth. To achieve these goals, some general requirements of artificial SEIs need to be considered. First, the artificial SEI structure should be uniform. Otherwise, the SEI layer may cause nonuniform ion flux, resulting in nonuniform deposition and dendrites [96,97]. In addition, the SEI should be physically, chemically and electrochemically stable. Moreover, the SEI should have enough strength and toughness to withstand the plating/stripping and dendrite growth. Finally, the SEI needs to remain adherent and intact during cycling.

Three categories of artificial SEIs are designed based on the material types, including an inorganic SEI, an organic SEI and a composite SEI.

The effect of the lithium-ion diffusion rate on dendrite growth has previously been investigated [98,99]. Although the mechanism of the effect is not fully explained, the phenomenon is demonstrated by experiments and simulation. The diffusion control processes prefer dendritic lithium deposition, while reaction-controlled processes tend toward spherical lithium deposition. Therefore, improving ionic conductivity in SEIs can maintain lithium deposition under reaction-controlled conditions and lead to a dense lithium deposition morphology. Compared to organic compounds, inorganic compounds typically have higher ion conductivity. For example, inorganic components such as Li_2_S and Li_3_N have very high ionic conductivity, which can enhance the ion diffusion rate and promote spherical lithium deposition. In addition, inorganic compounds usually have better hardness. Hence, inorganic-rich components of the SEI have high mechanical strength, which can mechanically suppress the growth of dendrite.

**Figure 5:**
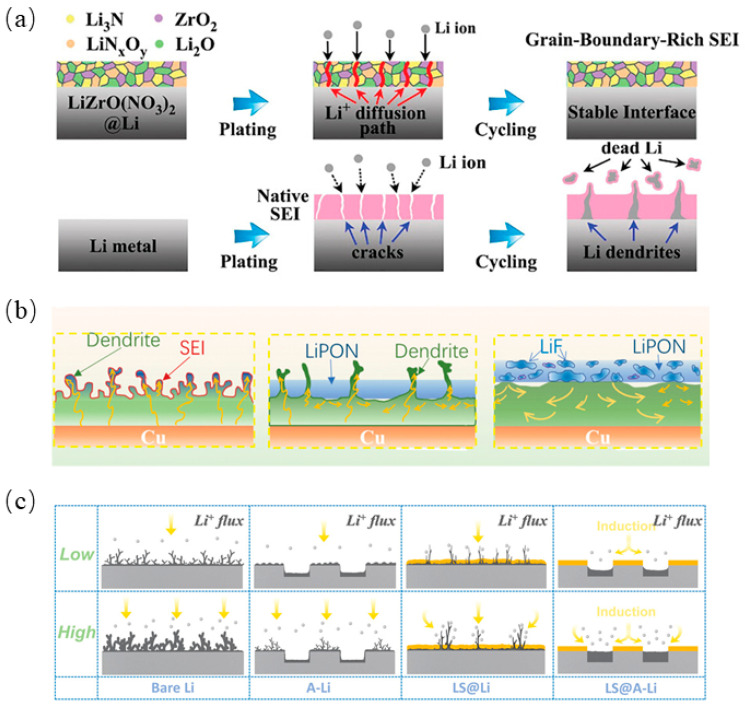
(**a**) Schematic illustration of Li stripping/plating behavior for the bare Li and LiZrO(NO_3_)_2_@Li anodes [100], (**b**) Schematic illustration of the lithium electrodeposition behavior in bare Cu, LiPON/Cu, and LiF–LiPON/Cu electrodes [102], (**c**) Schematic illustration of different Li deposition behavior of bare Li, A-Li, LS@Li, LS@A-Li at low and high current densities, respectively [103].

Li_3_N-based artificial SEI layers were constructed using the spontaneous reaction of lithium metal with zirconyl nitrate (ZrO(NO_3_)_2_); they consisted of ZrO_2_, Li_2_O, Li_3_N, and LiN_x_O_y_, with abundant grain boundaries, as shown in Figure 5a [100]. Since the diffusion of Li ions along the grain boundaries is faster than that of the bulk phase [101], the SEI can provide a rapid pathway for Li ions. Based on magnetron co-sputtering technology, LiF nanocrystals could be embedded in an LiPON inorganic amorphous matrix. After the insertion of LiF, the artificial SEI has high ionic conductivity and stronger mechanical stability, as shown in Figure 5b [102]. Another artificial SEI protective layer with Li_2_S_2_ as the main component was constructed on the top layer of an array-structured Li foil, as shown in Figure 5c [103]. Li_2_S/Li_2_S_2_ was homogeneously deposited on the top of Li foil via low-temperature selective vaporization Li_2_S_6_, while an Li_2_S layer with low electrical conductivity resulted in SEI deposition on the bottom layer of the Li foil. The uniform Li_2_S_2_ artificial SEI could protect the electrode–electrolyte interface and inhibit the growth of lithium dendrites, as well as provide a fast path for lithium-ion transport.

Although the inorganic SEI has high ionic conductivity and hardness, which is good for suppressing dendrite growth, the main disadvantage of inorganic compounds is the low flexibility, which leads to the failure of resisting the volume changes on the electrode during ion plating/stripping. On the other hand, organic polymeric components typically have high elasticity and flexibility, which can accommodate the volume changes of the electrode. Therefore, organic compounds are also considered to be used in artificial SEIs to achieve long-life batteries [73].

The preparation of organic lithium carboxylate SEI layers, which occurs through the in situ spontaneous reaction of lithium metal with carboxylic acid, has been reported [104]. Lithium carboxylate has a low Young’s modulus and high flexibility, which enables it to accommodate the volume changes of the electrode. In addition, it is found that alginate-based SEI films can be formed by using alginic acid and lithium hydroxide [105]. Alginate has stable chemical properties and superior ion transport properties, which can reduce side reactions and enhance the cycling stability of batteries.

A dynamic SEI reinforced by an open-architecture metal–organic framework (OA-MOF) film has been proposed, which leverages the elastic volumetric changes of three-dimensional lithiophilic sites for self-regulation. The self-adjusting distribution of lithiophilic sites on vertically grown Cu_2_(BDC)_2_ nanosheets enables a uniform Li-ion flux, precise control over Li mass transport, and compact lithium deposition [106]. A robust artificial SEI film with biomimetic ionic channels and enhanced stability is proposed, incorporating a ClO_4_^–^-functionalized metal–organic framework (UiO-66-ClO_4_) and a flexible lithiated Nafion (Li-Nafion) binder. The strong electronegativity and lithium affinity of ClO_4_^–^ groups, anchored within UiO-66 channels, establish a highly efficient single-ion conducting pathway, a high Li^+^ transference number, and superior ionic conductivity. This structure effectively inhibits detrimental reactions between the lithium metal and the electrolyte, while regulating rapid and uniform Li^+^ flux. Further reinforced by the flexible Li-Nafion binder, the UiO-66-ClO_4_/Li-Nafion (UCLN) composite film exhibits remarkable mechanical strength, suppressing lithium dendrite formation and ensuring the long-term structural stability of the lithium metal anode during cycling [107]. Two frameworks functionalized with -NH_2_ and -CH_3_ groups have been utilized as fillers in polyethylene oxide (PEO) composite solid electrolytes, with their catalytic roles in lithium formation at the SEI interface being investigated. First-principles calculations elucidate the LiF-rich SEI formation mechanism, demonstrating that ZIFs-NH_2_ significantly elongates the C–F bond in TFSI⁻ compared to ZIFs-CH_3_, thereby facilitating bond cleavage and enhancing LiF production [108].

**Figure 6:**
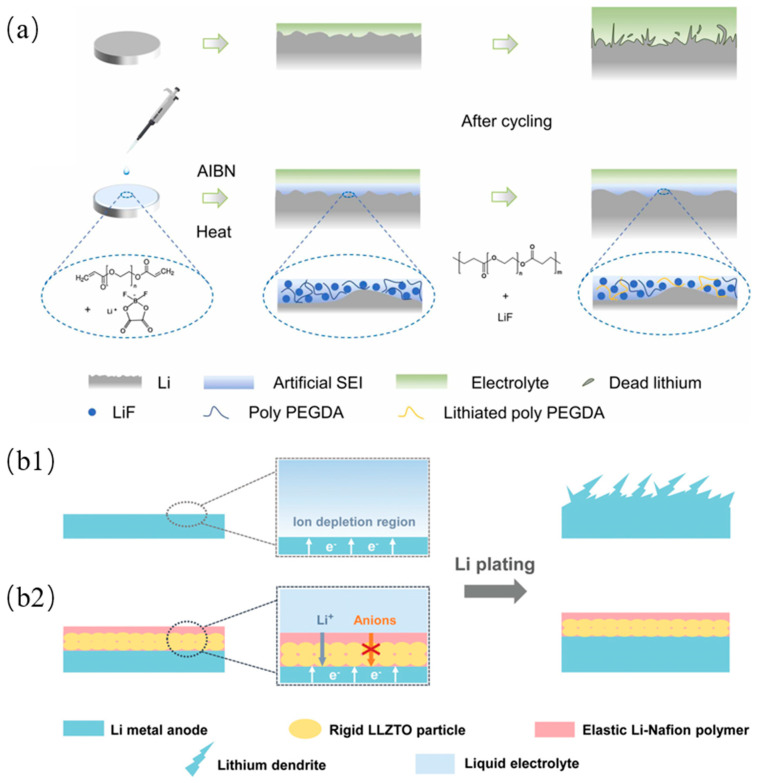
(**a**) Schematic illustration of organic-inorganic composite SEI preparation through in-situ polymerization and the corresponding Li deposition behaviors of bare Li and modified Li [110]. (**b1**) Schematic illustrations of different Li deposition patterns. (**b1**) The space charge region induced by anion depletion will impose a strong electric field at the vicinity of bare Li, leading to dendritic Li deposits. (**b2**) After incorporating the single-ion-conducting LLZTO/Li-Nafion coating composed of rigid LLZTO and elastic Li-Nafion, a uniform and compact Li plating behavior can be obtained [112].

However, most organic-based SEI films lack mechanical strength and have low ionic conductivity. Therefore, the pure organic SEI cannot meet the requirement for advanced batteries. As a result, the development of an organic–inorganic composite SEI, which combines the advantage of both materials to enhance SEI performance, is now attracting researchers’ attention. It is reported that an organic–inorganic composite SEI with both mechanical strength and flexibility, based on reactive polymers as SEI precursors, has been synthesized [109]. This composite SEI consists of polymeric lithium salts in LiF and graphene oxide (GO) nanosheets, and it helps to inhibit dendrite growth, prevent electrolyte decomposition, and promote efficient lithium deposition. Another organic–inorganic composite SEI is reported to have been synthesized via the in situ polymerization of precursors composed of poly(ethylene glycol) diacrylate (PEGDA) and LiDFOB, as shown in Figure 6a [110]. PEGDA can provide good mechanical strength, and the decomposition of LiDFOB provides a good Li^+^ transport pathway and better chemical stability. The high mechanical strength and good flexibility help the SEI accommodate volume changes during cycling.

In addition, anion migration blocks the transport of cation ions, which causes the slow diffusion of cation ions and the depletion of the anions, resulting in a strong electric field that promotes dendrite growth. A single-ion conducting artificial SEI with a 3D crosslinked network has been prepared by using pentaerythritol tetrakis (2-mercaptoacetate) (PETMP) and lithium bis(allylmalonato)borate (LiBAMB) in a thiol–ene click reaction [111]. The migration of the anion was limited due to the covalent binding of the BAMB anion. Therefore, only lithium ions can be transported in the 3D cross-linked network. Furthermore, the weak electrostatic interaction between the off-domain sp^3^ boron anions and the lithium ion improves the conductivity of the SEI. Another single-ion conducting SEI was prepared by using Li_6.4_La_3_Zr_1.4_Ta_0.6_O_12_ (LLZTO) as the base layer and Li-Nafion as the top layer, as shown in Figure 6b [112]. Moreover, LLZTO has high mechanical strength and Li-Nafion has high elasticity, which inhibits dendrite formation.

The SEI on the graphite anode and metallic Li anode attracts the majority of the attention. Graphite was the first commercialized and widely used anode in Li-ion batteries, while in pursuit of high capacity, researchers are increasingly focusing on metallic lithium anodes. The co-intercalation of lithium ions and solvent molecules leads to the exfoliation of the graphite anode. The failure mechanism requires the SEI on graphite to adjust the volume change as well as block the intercalation of solvent molecules. However, the capacity of the graphite anode limits further improvement. The study of the SEI on graphite focuses on explaining the failure mechanism rather than the applications. On the contrary, metallic lithium metal is believed to be the ideal anode. The main concern hindering its commercial application is dendrite growth. Although the formation mechanism of the SEI on lithium is also discussed, the ultimate goal is to construct a stable SEI on a metallic lithium anode that suppresses the growth of dendrite and eliminates fire hazards.

## 4. SEI in Mg-Ion Batteries

Mg metal is probably the most promising anode material due to its low standard potential (−2.37 vs. SHE) and dendrite-free characteristic [113,114]. However, the formation of a passivating surface film rather than an Mg^2+^ conducting SEI on the Mg anode surface, which hinders the transport of Mg^2+^ ions, has always restricted the development of MIBs [115]. Thus, constructing an SEI with high ion conductivity becomes one of the most critical bottlenecks hindering the development of magnesium-ion batteries. Similar to lithium-ion batteries, electrolyte modification and artificial SEI construction are considered to help in the construction of a stable SEI.

In 2000, the first prototype electrolyte for an MIB was developed based on a Grignard reagent. The chemical formula can be expressed as RMgX (R may be alkyl or aryl; X is Cl, Br, or other halides). This electrolyte allows for reversible Mg deposition-stripping while preventing the formation of a passivation film [116]. However, due to the presence of halides, Grignard reagents are corrosive, particularly to current collectors [117,118], which leads to stability issues over time. In addition, Grignard-based electrolytes typically have a limited electrochemical stability window, which restricts their use with high-voltage cathodes. Moreover, Grignard-based electrolytes are highly sensitive to air and moisture, which makes handling and manufacturing challenging. Researchers continue to work hard to overcome these disadvantages, which limit the overall energy density of the battery [119,120].

To address the interfacial passivation of Mg metal anodes in electrolytes, researchers focus on changing salts and adding additives into the electrolytes. Mg salt (MgFPA), which has an Al(III)-centered anion Al(O_2_C_2_(CF_3_)_4_)_2_, abbreviated as FPA, with a large radius could induce solvent coordination [121]. The LUMO energy level of the FPA anion was decreased after the coordination between the FPA anion and the THF solvent molecule. As a result, a thin and stable SEI with superior Mg^2+^ conductivity was formed on the Mg metal anode in the MgFPA/Tetrahydrofuran electrolyte. From the same research group, another electrolyte consisting of Li [B(hfip)_4_]/DME (LBhfip/DME) (hfip=OC(H)(CF_3_)_2_) was also synthesized [122]. In the initial cycling process, Mg^2+^ would be introduced into LBhfip/DME, forming a hybrid Li^+^/Mg^2+^ electrolyte, which could conduct Mg^2+^ in subsequent cycles. Moreover, an Li-containing SEI was formed on the Mg metal anode, which could not only effectively conduct Mg^2+^ but also prevent the continuous corrosion of the Mg metal anode by liquid electrolytes. A stable SEI formed on the Mg metal surface by adding an Mg(BH_4_)_2_ additive to the Mg [B(hfip)_4_]_2_/DME electrolyte. In this electrolyte system, the Mg(BH_4_)_2_ additive could remove the passivation layer on the Mg metal surface and then enhance the Mg storage performance [123].

Introducing a non-passivating anionic additive—reduced perylene diimide ethylenediamine (rPDI)—into the electrolyte enables the rapid and reversible deposition/dissolution of Mg within a simple non-nucleophilic electrolyte system. The rPDI additive demonstrates higher adsorption energy on the Mg surface compared to TFSI salt, while PDI preferentially adsorbs onto the Mg surface. Acting as a protective anion, it prevents TFSI decomposition and Mg anode passivation by forming a solid electrolyte interface (SEI) layer on the surface of the Mg anode that facilitates Mg^2+^ conduction, which provides a straightforward and effective strategy for integrating magnesium anodes with diverse organic and conversion materials in non-nucleophilic electrolyte environments [124]. An ideal solvent system for developing high-performance MIBs must simultaneously exhibit high solubility for magnesium salts and provide effective protection for magnesium metal. Meeting these criteria with a single solvent proves challenging. Diethyl malonate (DEM) is frequently utilized to enhance the dissociation of magnesium salts through multidentate coordination with Mg^2+^ cations, which facilitates ion dissociation and migration under an electric field. In contrast, cyclic ethers aid in the formation of protective films on metal anodes via ring-opening reactions. By harnessing the advantages of both types of solvents, a solution composed of non-nucleophilic Mg(TFSI)_2_-MgCl_2_ in DME, dispersed within a non-fluorinated, weakly coordinating solvent such as THF, can effectively prevent the decomposition of DME while establishing a stable SEI. The dimethyl ether solvation shell, characterized by its migratory components, is surrounded by weakly coordinated cyclic ethers, which facilitates SEI formation without inducing ion binding. Compared to DME alone, the mixed solvent system exhibits a slightly reduced solvation capacity, thereby ensuring compatibility with Mg^2+^ without compromising coordination efficiency [125].

To avoid the issues associated with Grignard-based liquid electrolytes, many new liquid electrolytes have been proposed, such as organoborate-based electrolytes, borohydride-based electrolytes, nitrogen-containing electrolytes, magnesium aluminate chloride complex electrolytes, Mg(TFSI)_2_-based electrolytes, and ionic liquid electrolytes [116,126]. However, there is still no mature electrolyte system for Mg-ion batteries.

Hence, constructing the artificial SEI on the anode surface can bypass the issues inherent in the electrolyte. An artificial SEI layer can be formed on the surface of the magnesium anode through a displacement reaction involving ZnCl_2_ and metallic Mg. Due to the higher equilibrium electrode potential of zinc compared to magnesium (E _Zn_^2+^/_Zn_ = −0.763 V vs. SHE, E _Mg_^2+^/_Mg_ = −2.3 V vs. SHE), ZnCl_2_ readily undergoes a substitution reaction with metallic Mg (ZnCl_2_ + Mg → MgCl_2_ + Zn). This reaction is exothermic (ΔH_rxn_ = −102 kJ/mol), indicating its spontaneity. As the reaction proceeds, an SEI layer incorporating an MgZn_2_ alloy is formed. This alloy induces a significant number of tilted grain boundaries on the magnesium surface, which symmetrically lowers the reaction barriers for both anode and cathode processes, thereby enhancing the exchange current density [127].

**Figure 7:**
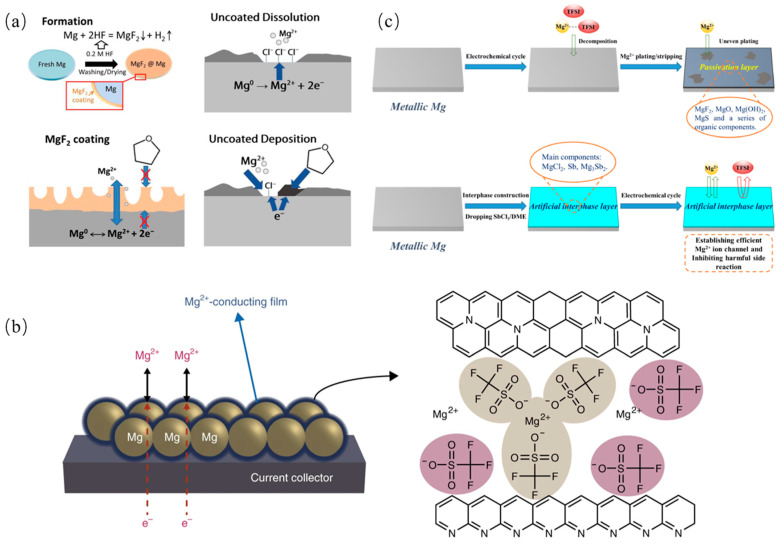
(**a**) Schematic illustration of the formation process of the MgF_2_ surface coating and surface chemistry of coated/uncoated Mg anode [128]. (**b**) Schematic of a Mg powder electrode coated with the artificial Mg^2+^-conducting interface, and the proposed structure for the artificial Mg^2+^-conducting interface [129]. (**c**) Schematic diagrams of electrochemical behavior of pristine Mg anode and modified Mg anode [134].

Similarly, inorganic, organic and composite SEIs are all investigated by researchers. SEIs containing magnesium fluoride (MgF_2_), which control the reaction of the Mg metal surface with hydrofluoric acid (HF), can not only suppress the side reaction with the electrolyte but also allow for the generation of Mg^2+^ transport between electrodes and electrolytes, as shown in Figure 7a [128]. Another artificial interface composed of Mg powder, Mg(CF_3_SO_3_)_2_, polyacrylonitrile (PAN), and carbon black is compatible even within the carbonate-based electrolyte [129]. Figure 7b illustrates that this artificial interface delivers a moderate ionic conductivity and a low electronic conductivity, which could promote Mg stripping/plating reversibility and prevent electrolyte reduction. Moreover, a 3D SEI with Mg_3_Bi_2_ scaffolds on the Mg anode surface is designed to avoid continuous passivation and to mitigate the degradation of the SEI [130]. Bi is a good candidate of anodes due to its excellent electrochemical properties, despite its price [131]. The 3D Mg_3_Bi_2_ scaffolds possess large specific surface areas, thereby greatly reducing the current density, and the continuous passivation is avoided. Another Bi-based artificial protecting layer on Mg metal anodes was designed via a facile solution strategy [132,133]. In Mg(TFSI)_2_-based electrolytes, this artificial layer could avoid parasitic reactions, while in APC electrolytes, this protective layer could facilitate Mg-atom adsorption and diffusion. An Sb-based artificial interface layer mainly containing MgCl_2_ and Mg_3_Sb_2_ endows the significantly improved interfacial kinetics and electrochemical performance of the Mg anode, which significantly reduces overpotential and enables swift Mg^2+^ migration, as shown in Figure 7c [134]. By simply soaking Mg foil in a tetraethylene glycol dimethyl ether solution containing LiTFSI and AlCl_3_, an artificial SEI is constructed, which could mitigate Mg passivation in Mg(TFSI)_2_/DME electrolytes. This approach is also extended to Mg(ClO_4_)_2_/DME and Mg(TFSI)_2_/PC electrolytes to achieve reversible Mg plating and stripping, which also ensures the interfacial resistance of the cells, with the SEI protected by Mg two orders of magnitude lower than bare Mg [135]. An artificial SEI coating composed of MgBr_2_, MgI_2_, and Sb on the Mg surface through an off-site displacement reaction can enhance the reversible deposition and dissolution of Mg^2+^, significantly reduce overpotential, and achieve superior specific capacity retention with lower polarization across the entire battery. Calculations reveal that the diffusion energy barriers for Mg^2+^ in Sb, MgBr_2_, and MgI_2_ are 1.72 eV, 0.41 eV, and 0.18 eV, respectively, indicating that the SEI layer effectively facilitates ion conduction [136].

**Figure 8:**
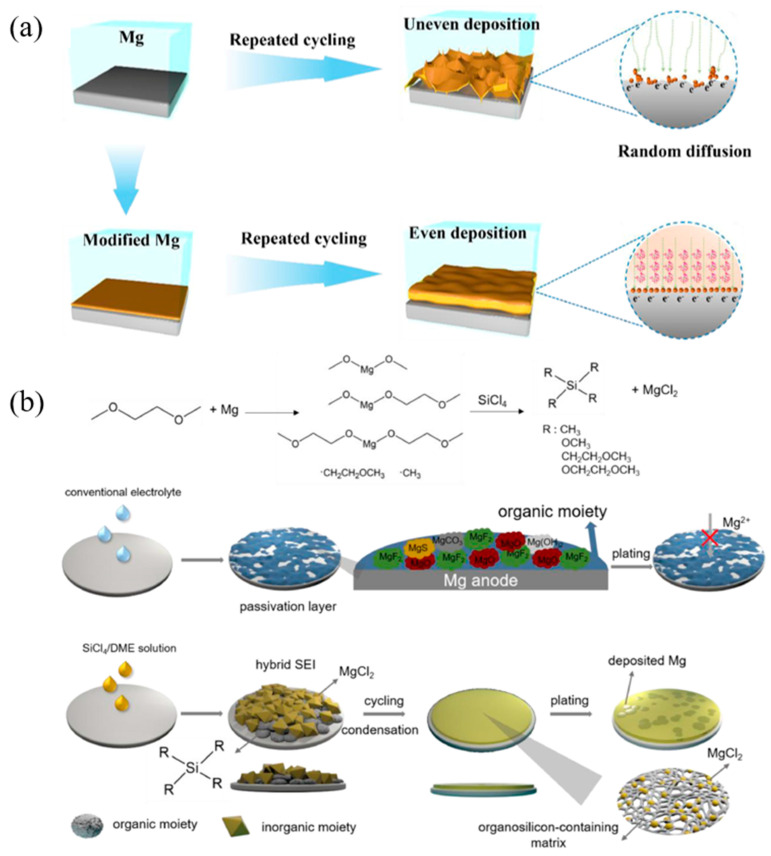
(**a**) Scheme illustrations for interface reactions with and without PA artificial SEI layers on the Mg surface [137]. (**b**) Possible mechanism on the solvent-assisted additive displacement strategy and the formation of inorganic/organic hybrid SEI after the contact of Mg surface with SiCl_4_/DME solution. Schematic diagrams of SEI evolution and Mg deposition manner based on bare Mg electrode and Mg-Si electrode in conventional electrolyte [139].

An organic SEI is designed using the dynamic self-assembly coordination of phytic acid (PA) with Mg^2+^. Figure 8a shows that the 3D porous channel structure of the PA skeleton could regulate ion flux and efficiently homogenize the distribution of Mg^2+^ [137]. Through the drip-coating method, a polymer-alloy composite SEI is constructed on the Mg surface, with a polymerized tetrahydrofuran (PTHF) network cross-linked with Mg-Cl and Sn-Cl complexes and a metallic layer with Sn and Mg-Sn domains from top to bottom. The upper PTHF network with rich Mg-Cl moieties facilitates fast Mg-ion transport and its flux homogenization, while the lower Sn-based magnesophilic domains provide abundant Mg deposition sites with low nucleation and migration barriers, ensuring uniform Mg plating and stripping [138]. Using a solvent-assisted additive displacement strategy, another composite SEI with a low surface composed of an MgCl_2_-rich top layer and an organosilicon-dominated bottom layer is designed, which can withstand long-term anode cycling and permanently protect the Mg anode against passivation in conventional electrolytes, as shown in Figure 8b [139]. An SEI composed of amorphous an MgCl_2_@polymer on the Mg-metal surface is prepared by the in situ chemical reaction of metallic Mg with H_3_PO_4_ and SiCl_4_ in sequence, which can effectively inhibit electrolyte decomposition and facilitate Mg^2+^ transport [136]. Incorporating Aquivion and SPEEK polymers into polyacrylonitrile (PAN) can also create an organic artificial SEI on Mg metal foil. Unlike inorganic SEI layers, this polymer/ionic organic SEI layer mitigates severe surface reactions that lead to the formation of macroscopic pores and localized coating failures during cell operation. Consequently, the Mg-S battery constructed using this approach exhibits high initial-cycle Coulombic efficiency and maintains a high discharge capacity over cycling [136].

Moreover, the Mg-Li alloy exhibits unique SEI characteristics in magnesium-based battery systems. Due to the electrochemical differences between magnesium and lithium, the alloying process on the electrode surface leads to the formation of a more stable SEI layer. Compared to pure magnesium electrodes, the SEI layer on Mg-Li alloys typically shows enhanced stability and ionic conductivity. This is primarily because the SEI layer contains lithium/magnesium compounds, which effectively prevent further side reactions between magnesium ions and the electrolyte, thereby reducing corrosion and improving the cycling stability. Furthermore, the incorporation of lithium modifies the interfacial structure of the magnesium electrode, facilitating the reversible insertion and extraction of magnesium ions and ultimately enhancing the overall electrochemical performance. A robust SEI layer that develops on the surface of the Li-Mg alloy anode can effectively mitigate the side reactions and preserve surface smoothness throughout cycling. The Li-deficient Li-Mg alloy forms a porous skeletal structure that facilitates both electron and Li-ion conduction, ensuring the structural integrity of the anode during the Li/Mg stripping/plating process [140]. A magnesium-lithium alloy has been identified as a passivation-free anode, effectively preventing passivation reactions through a substitution mechanism between lithium in the alloy and magnesium ions in the electrolyte. This alloy anode demonstrates significantly enhanced interfacial reaction kinetics, with an impedance reduction of five orders of magnitude compared to that of a magnesium anode [141,142].

In summary, the SEI construction strategies can be classified as solvent shell structure alteration, electrolyte additives, electrode modification, artificial SEI, in situ SEI formation via spontaneous reactions, etc. The characteristics of each method are summarized in Table 1. The challenges of the construction strategies limit their wide utilizations and become the focus of the SEI research.

## 5. Summary and Perspectives

This review aims to clarify the basic understanding of the electrochemical processes, influencing factors, and multiple approaches to constructing a stable SEI. The basic formation mechanism of SEIs is now explained well by molecular orbital theory, while the explanation of redox reaction processes remains ambiguous. The intrinsic properties of electrolytes and electrodes, as well as external factors such as temperature and current density, affect formation and evolution, which also signals to researchers to construct SEIs with good stability and ionic conductivity by modifying electrolytes and electrodes or controlling experimental conditions. The SEI in two battery systems—Li-ion batteries and Mg-ion batteries—is reviewed in detail.

The research history of Li-ion batteries is much longer than that of Mg-ion batteries. Most of the SEI construction ideas in Mg-ion batteries come from the work of Li-ion batteries, which makes the SEI construction strategies in the two battery systems very similar, including electrolyte modification and artificial SEI construction. There are two methods widely used in both Li-ion batteries and Mg-ion batteries. Electrolyte modification not only helps in constructing an ideal SEI but also improves the intrinsic property of the electrolyte. Since the electrolyte system in Li-ion batteries is quite mature, the focus of electrolyte modification is on the additive. On the contrary, in Mg-ion batteries, there is still no satisfactory electrolyte, and the focus shifts to developing an ideal electrolyte system rather than additives. On the other hand, in situ artificial SEI construction attracts more and more attention due to its good adhesion and customization, despite the high cost and mass production problem, which hinders commercial applications.

However, the functionality of SEI construction in the two batteries is different, which causes the strategic focus to differ. The main purpose of the SEI in an Li-ion battery is suppressing dendrite growth, while in an magnesium-ion battery, the focus of SEI construction is to suppress the passivation film and improve ion transport. Therefore, when constructing the SEI in an Li-ion battery, the stiffness of the SEI is a primary concern. It is preferred to construct inorganic compounds and ceramics in the SEI. On the contrary, when constructing the SEI in an Mg-ion battery, the key point is to improve the ionic conductivity of the interface. Since the Mg anode is believed to be a dendrite-free anode and simple Mg salts have lower ionic conductivity than their Li salt counterparts, polymer and other organic compounds are used to construct the SEI. Another difference is that Mg alloys are considered not only as anodes but also as components of the SEI due to good conductivity and stability, while few Li alloys are discussed in the literature. In summary, the SEI construction strategies and technologies are quite similar in the two battery systems. The different concerns and requirements lead to different choices of materials and compositions of SEIs.

Although considerable research has been conducted, there is still no satisfactory way to construct a mature SEI due to the following challenges. The understanding of SEI formation, in terms of the reaction kinetics and mechanisms, composition, and role in battery performance is insufficient. Moreover, a deeper understanding of the fluctuating local current density distribution regarding ion deposition nucleation and further growth is lacking. The characterization technologies, especially the in situ characterization, still has many shortcomings, which hinders its wide application in the study of the SEI. For example, the current distribution and electric field gradient in an in situ electrochemical cell are different from those in coin batteries or commercial batteries. In situ electrochemical cells are limited by their electrolyte volume, which cannot measure the size effect of the electrodes on the electric field distribution in the electrolyte. Hence, in situ characterization currently has difficulty directly reflecting the same process in coin batteries and commercial batteries.

On the other hand, these issues also guide us in overcoming the challenges. Several future research directions may be proposed as follows.

QC/AIMD/DFT calculations should be promising methods for SEI modeling. With the optimization of computing software and the improvements in computing capability, the multi-scale and full-scale modeling of SEIs have been gradually implemented. The influence of geometry on local current density and deposition behavior can be further investigated. The current is affected by the uneven geometry, causing inconsistent current density, which significantly impacts deposition behavior. Thus, quantitatively analyzing the current at the interface is essential to understanding deposition behavior.

New advanced in situ characterization methods can be further developed. The size of current in situ electrochemical cells limits the full understanding of the performance of materials. Local performance or behavior can even mislead our understanding. The study of the impact of multi-physics coupling on material performance is also limited by the current in situ characterization hardware. Advanced in situ characterization methods can ensure the accuracy of experimental results and provide new insights into the SEI working mechanism.

In this review, we address the issues related to the SEIs of LIBs and MIBs. Meanwhile, the factors affecting SEI construction are explained. The strategies for constructing the SEI on the anode are also discussed through case studies. In view of the interfacial complexity, only small aspects of these issues have been solved under specific conditions. Many phenomena remain unresolved and not yet fully understood. The treatment of the interface and the characterization of the SEI can provide adequate explanations and help construct better SEIs in batteries.

## Figures and Tables

**Table 1:** The characteristics of SEI construction strategies.

Method	Description	Key Advantages	Challenges	Impact on SEI Performance
Solvent Shell Structure Alteration [22,23]	Altering solvent shell structures around ions in the electrolyte to form a more stable SEI	Improved control over SEI composition	Requires precise solvent system design to balance ion transport and SEI stability	Alters SEI formation by controlling ion solvation, leading to more uniform and protective SEI layers
Electrolyte Additives [89,90]	Introducing additives to the electrolyte to enhance SEI formation	Low-cost, easily adaptable	Additives may decompose, leading to impurities or unwanted reactions	Enhances stability and conductivity by promoting favorable SEI compounds
Electrode Modification [18,115]	Interlayer the electrodes to reduce the volume change of the electrode	Enhances electrode stability	Requires complex processing	Produces a stable, uniform SEI by modifying the electrode surface to encourage better electrolyte interaction
Artificial SEI [135,139]	Pre-fabricated SEI films applied to the electrode to control initial SEI formation	Highly tunable and can be designed for specific ion transport properties	Complicated fabrication process	Artificial SEIs can be customized for high stability and conductivity, suppressing dendrites and improving performance
In situ SEI Formation via Spontaneous Reactions [136]	SEI forms spontaneously during cycling from electrolyte decomposition	Simple process, naturally conforms to electrode surface	Often uncontrolled and result in non-uniform SE	In-situ formed SEI layers may be thicker and less uniform, resulting in slower ion transport
